# Changes in the prefronto-thalamic tract following cranioplasty: Case reports

**DOI:** 10.1097/MD.0000000000025350

**Published:** 2021-04-09

**Authors:** Eun Bi Choi, Chul Hoon Chang, Sung Ho Jang

**Affiliations:** a Department of Physical Medicine and Rehabilitation, College of Medicine, Yeungnam University; b Department of Neurosurgery, College of Medicine, Yeungnam University, Daegu, Republic of Korea.

**Keywords:** cognitive impairment, cranioplasty, diffusion tensor imaging, prefronto-thalamic tract, stroke

## Abstract

**Rationale:**

The prefrontothalamic tract (PTT) injury is associated with various neuropsychological impairments including cognitive impairment. We report on three women with hemorrhagic stroke who showed changes in the PTT following cranioplasty (C/P) using diffusion tensor tractography (DTT) images.

**Patient concerns:**

The 3 women with hemorrhagic stroke showed reductions of cognitive impairment following C/P. Mini-Mental State Examination scores (MMSE) were increased by 7-, 8-, and 5-point in patient 1, 2, and 3, respectively, after C/P compared with the patients’ pre-C/P MMSE scores.

**Diagnosis:**

Three patients were diagnosed with spontaneous intracerebral hemorrhage. Three patients underwent C/P using auto-bone at 7 (patient 1 and 3) and 13 (patient 2) weeks after onset.

**Interventions:**

Diffusion tensor imaging data were acquired within 3 days before and 21 days after C/P.

**Outcomes:**

The pre-C/P DTT results showed non-reconstruction of the dorsolateral prefrontal cortex (DLPFC; patient 2 and 3) on the contralateral operation (contra-OP) side and orbitofrontal cortex (OFC; patient 3) on both sides, but those were reconstructed on post-C/P DTT. Except for the contra-OP side OFC of patient 2, all fractional anisotropy values decreased on post-C/P DTT compared with pre-C/P DTT. The mean diffusivity values of the VLPFC and OFC were higher on post-C/P DTT except for the contra-OP side VLPFC of patient 1 and contra-OP side OFC of patient 2. The voxel numbers also increased except for the contra-OP side VLPFC of patient 1.

**Lessons:**

We demonstrated structural changes in the PTT along with concomitant reductions of cognitive impairments following C/P in 3 women with hemorrhagic stroke using DTT. The DTT changes suggest that C/P can affect the state of the PTT on both the OP and contra-OP sides. However, the limitation that DTT analysis may underestimate or overestimate fiber tract status due to regions of fiber complexity and crossing fiber should be considered.

## 1 Introduction

Cranioplasty (C/P) for the restoration of cranial defects is a surgical operation that incorporates a bone piece from the patient or a synthetic material. C/P is performed not only for the repair of skull defects but also for cosmetic purposes or to afford protection against neurological deterioration following craniectomy.^[1–4]^ Previous studies have reported reductions in cognitive impairments following C/P.^[1,5,6]^ The mechanisms of reduction of cognitive impairment following C/P have been related to improvements to cerebral blood flow, cerebrospinal fluid hydrodynamics, and metabolic activity.^[7–10]^ However, the mechanisms have not yet been elucidated in detail.

The prefrontal cortex (PFC) is involved in numerous cognitive functions, and the main functions of the 3 subregions of the PFC are as follows: dorsolateral prefrontal cortex (DLPFC)—attention, planning, working memory, and mood control; ventrolateral prefrontal cortex (VLPFC)—deliberation for decision-making, behavior regulation, trial-and-error learning, and goal-directed behavior; orbitofrontal cortex (OFC)—decision-making and memory.^[11–14]^ Several studies have reported changes in the PFC can occur concurrently with improvement to cognitive impairment following C/P.^[15–20]^ The PFC receives profuse afferent fibers from the dorsomedial nucleus of the thalamus via the prefronto-thalamic tract (PTT),^[11–14]^ and PTT injury is associated with various neuropsychological impairments including cognitive impairment.^[11–14,21,22]^ Because the PTT cannot be fully discriminated from adjacent neural structures on conventional brain computed tomography (CT) or magnetic resonance imaging (MRI), precise estimation of the PTT has been limited in the live human brain. However, the development of diffusion tensor tractography (DTT), an extension of diffusion tensor imaging (DTI), enabled structural estimation and visualization of the PTT via its capacity to provide three-dimensional tract reconstruction.^[13,14,21,22]^

DTT has a unique advantage in determining changes in a neural tract by providing a configurational analysis of a three-dimensional reconstruction of a neural tract.^[13,14,21,22]^ In addition, structural changes to a neural tract can be determined by examining changes in three DTT parameters: fractional anisotropy (FA), mean diffusivity (MD), and voxel number (VN). The FA variable represents the state of white matter organization by indicating the degree of directionality and integrity of white matter microstructures, the MD variable indicates the magnitude of water diffusion of a neural tract, and the VN parameter indicates the number of voxels included in a neural tract, which is deemed representative of the total number of fibers within that tract.^[23–26]^ A few studies have used DTT results to show changes in neural tracts, including the corticobulbar and corticoreticulospinal tracts, following C/P.^[27,28]^ To date, no study on changes in the PTT following C/P has been reported.

In the present study, we report on three women with hemorrhagic stroke who showed changes in the DTT parameters of their PTTs that were concomitant with reductions of cognitive impairment following C/P.

## 2 Materials and methods

### 2.1 Subject

**Figure 1:**
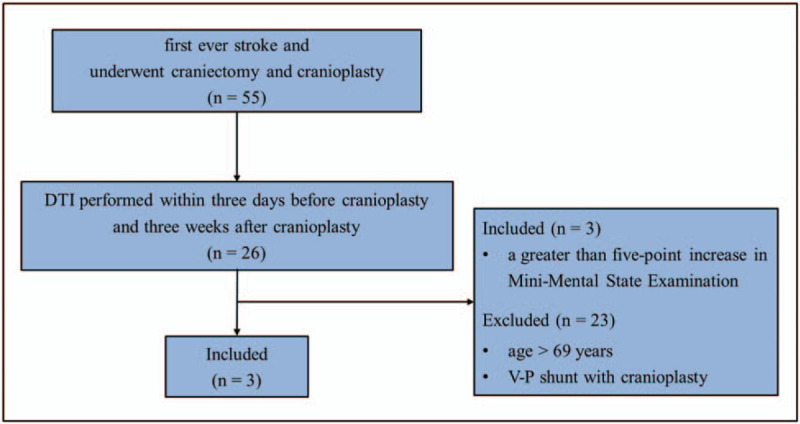
Flow diagram of the inclusion criteria, DTI = diffusion tensor imaging, V-P shunt = ventriculo-peritoneal shunt.

Three women with hemorrhagic stroke who showed reduction of cognitive impairments following C/P were recruited for this study (Table 1). The 3 patients were recruited according to the following inclusion criteria (Fig. 1): first-ever stroke, craniectomy for the management of brain hematoma or swelling performed at the neurosurgery department of a single university hospital, DTI performed within three days before C/P (pre-C/P) and 3 weeks after C/P (post-C/P), and a >5-point increase in Mini-Mental State Examination score (MMSE, full score: 30, cutoff score <24) after C/P.^[29]^ The institutional review board of the university hospital approved the study protocol, and the patients of the study provided signed informed consent (approval No. YUMC-2019–06-032).

**Table 1:** Demographic and clinical characteristics of the three patients.

	Patient 1	Patient 2	Patient 3
Sex/age, y	F/57	F/51	F/63
Lesion	SAH + ICH	ICH	SAH + ICH + IVH
Operation	Craniectomy + hematoma removal, C/P	Craniectomy + hematoma removal, C/P	Craniectomy + hematoma removal, C/P
Operation side	Rt	Lt	Rt
C/P from onset, wk	7	13	7
DTI, day^∗^			
Pre-C/P	1	2	3
Post-C/P	9	6	21
Change of MMSE^†^	+7	+8	+5

C/P = cranioplasty, DTI = diffusion tensor imaging, F = female, ICH = intracerebral hemorrhage, IVH = intraventricular hemorrhage, Lt = left, MMSE = Mini-Mental State Examination, Rt = right, SAH = subarachnoid hemorrhage.

∗ Days from diffusion tensor imaging to and from cranioplasty.

† Change in MMSE score from pre-C/P to post-C/P.

### 2.2 Diffusion tensor imaging

DTI data were acquired before and after C/P (pre-C/P: mean duration, 2 days before C/P; range, 1∼3 days; post-C/P: mean duration, 12 days after C/P; range, 6∼21 days) using a 6-channel head coil on a 1.5T Philips Gyroscan Intera (Philips, Best, Netherlands) with single-shot echo-planar imaging. For each of the 32 non-collinear diffusion sensitizing gradients, 63 contiguous slices were acquired parallel to the anterior commissure-posterior commissure line. Imaging parameters were as follows: acquisition matrix= 96 × 96, reconstructed to matrix = 192 × 192, field of view = 240mm × 240 mm, repetition time= 10,398 ms, echo time= 72 ms, parallel imaging reduction factor (SENSE factor) = 2, echo-planar imaging factor = 59, b = 1000 s/mm^2^, number of excitations = 1, and slice thickness = 2.5 mm.

### 2.3 Probabilistic fiber tracking

The Oxford Center for Functional Magnetic Resonance Imaging of the Brain (FMRIB) Software Library (www.fmrib.ox.ac.uk/fsl) was used to analyze diffusion-weighted imaging data used in the reconstruction of the PTT. Affine multiscale 2-dimensional registration was used to correct for head motion effects and image distortion due to eddy currents. Fiber tracking was performed using FMRIB Diffusion Software with the routine option (0.5 mm step lengths, 5000 streamline samples, curvature thresholds = 0.2) activated in a probabilistic tractography method. For analysis of the PTT, the seed region of interest (ROI) was placed on the mediodorsal nucleus, and target ROIs were on the DLPFC, the VLPFC on coronal images, and the OFC on axial images.^[13]^ Results of fiber tracking were visualized using 5000 samples generated from the seed voxel with a threshold of two streamlines through each voxel used for analysis. The FA, MD, and VN values of the DTT-reconstructed PTT were obtained for both the C/P operated (OP) and nonoperated (contra-OP) hemispheres.

## 3 Case presentation

### 3.1 Patient 1

**Figure 2:**
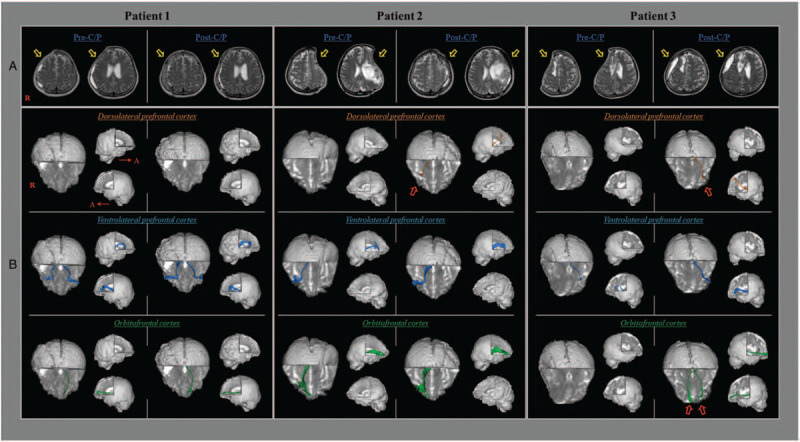
(A) T2-weighted brain magnetic resonance images of three women with hemorrhagic stroke before (pre-C/P) and after cranioplasty (post-C/P) (yellow arrows: OP side). (B) Diffusion tensor tractography (DTT) of the dorsolateral prefrontal cortex, the ventrolateral prefrontal cortex, and the orbitofrontal cortex of the prefronto-thalamic tract. The non-reconstructed dorsolateral prefrontal cortices of patients 2 and 3 on the contra-OP side and the orbitofrontal cortex of patient 3 on both sides on pre-C/P DTT were able to be reconstructed on post-C/P DTT (red arrows).

The 57-year-old female patient was diagnosed with spontaneous subarachnoid hemorrhage due to a ruptured aneurysm of the right middle cerebral artery and spontaneous intracerebral hemorrhage at the right basal ganglia (Table 1, Fig. 2A). The patient underwent decompressive craniectomy and hematoma removal at the neurosurgery department of a university hospital one day after stroke onset, and C/P using auto-bone was performed seven weeks after onset. After C/P, her cognitive impairment improved seven points on MMSE (MMSE scores: 22 at 1 day before C/P → 29 at 9 days after C/P). DTT was not able to reconstruct the DLPFC in either hemisphere before or after C/P (Fig. 2-B). On post-C/P DTT, the FA values of the VLPFC on the OP side and the OFC on the contra-OP side were lower than on pre-C/P DTT, whereas the post-C/P MD and VN values of these neural tracts were higher. By contrast, the FA and VN values of the VLPFC on the contra-OP side were lower (Table 2).

**Table 2:** Comparison of diffusion tensor tractography parameters and Mini-Mental State Examination scores for regions of the prefronto-thalamic tract pre- and post-cranioplasty in three women with hemorrhagic stroke.

	Patient 1				Patient 2				Patient3			
	Pre-C/P		Post-C/P		Pre-C/P		Post-C/P		Pre-C/P		Post-C/P	
	OP side (Rt)	Contra-OP side (Lt)	OP side (Rt)	Contra-OP side (Lt)	OP side (Lt)	Contra-OP side (Rt)	OP side (Lt)	Contra-OP side (Rt)	OP side (Rt)	Contra-OP side (Lt)	OP side (Rt)	Contra-OP side (Lt)
DLPFC												
FA	—	—	—	—	—	—	—	0.35	—	—	—	0.35
MD	—	—	—	—	—	—	—	0.80	—	—	—	0.76
VN	—	—	—	—	—	—	—	249	—	—	—	412
VLPFC												
FA	0.36	0.37	0.33	0.35	—	0.33	—	0.32	—	0.37	—	0.35
MD	0.84	0.81	0.85	0.81	—	0.82	—	0.84	—	0.75	—	0.80
VN	325	709	801	586	—	703	—	1512	—	216	—	633
OFC												
FA	—	0.37	—	0.33	—	0.28	—	0.31	—	—	0.32	0.33
MD	—	0.82	—	0.87	—	0.89	—	0.86	—	—	0.84	0.78
VN	—	310	—	468	—	1720	—	1986	—	—	580	390
MMSE	22		29		NT		8		NT		5	

C/P = cranioplasty, Contra = contralateral, DLPFC = dorsolateral prefrontal cortex, FA = fractional anisotropy, Lt = left, MD = mean diffusivity, MMSE = mini-mental state examination, NT = not testable, OFC = orbitofrontal cortex, OP = operation, Rt = right, VLPFC = ventrolateral prefrontal cortex, VN = voxel numbers.

### 3.2 Patient 2

The 51-year-old female patient underwent craniectomy and removal of hematoma in the fronto-parietal lobes at the department of neurosurgery of the same university hospital (Table 1, Fig. 2A). She underwent C/P with auto-bone at 13 weeks after onset. Her MMSE score increased by eight points from the pre-C/P value (MMSE: not testable at 2 days before C/P → 8 points at 6 days after C/P). The DLPFC in both hemispheres was not reconstructed on pre-C/P DTT; in contrast, the DLPFC on the contra-OP side was reconstructed on post-C/P DTT (Fig. 2B). The FA value decreased, and the MD increased in the VLPFC on the contra-OP side, whereas the values of FA and MD for the OFC increased and decreased, respectively, on the contra-OP side. The VN values of the VLPFC and OFC increased on the contra-OP side (Table 2).

### 3.3 Patient 3

The 63-year-old female patient was diagnosed with spontaneous intracranial hemorrhage in the right frontal lobe, as well as subarachnoid and intraventricular hemorrhages (Table 1, Fig. 2A). The patient underwent craniectomy and removal of hematoma at the neurosurgery department of the same university hospital. C/P was performed using auto-bone at seven weeks after onset. The patient's post-C/P MMSE score increased by five points compared to the pre-C/P MMSE score (MMSE: not testable at 3 days before C/P → 5 at 21 days after C/P). The DLPFC and OFC were not reconstructed in either hemisphere on pre-C/P DTT. In contrast, the DLPFC on the contra-OP side and the OFC on both sides were reconstructed on post-C/P DTT (Fig. 2B). On post-C/P DTT, the FA value decreased, and the MD and VN values increased on the contra-O/P side of the VLPFC compared to the pre-C/P DTT results (Table 2).

## 4 Discussion

In this study, by using follow-up DTT assessments, changes in the DLPFC, VLPFC, and OFC were detected in three women with hemorrhagic stroke who, concurrent with the DTT changes, showed reductions of cognitive impairments following C/P. We observed the following 2 changes. First, DTT configurations changes: the pre-C/P DTT non-reconstructed contra-OP side DLPFC (patients 2 and 3) and both sides of the OFC (patient 3) were reconstructed on post-C/P DTT. Second, DTT parameter changes: FA values were lower on post-C/P DTT than on pre-C/P DTT except for the contra-OP side of the OFC in patient 2; MD values increased except for the contra-OP sides of the VLPFC of patient 1 and the OFC in patient 2; VN values increased except for the contra-OP side of the VLPFC in patient 1. The 3-dimensional reconstruction of the nonreconstructed DLPFC and OFC on post-C/P DTT indicates there was restoration of the injured or compressed PTT. In addition, recovery was indicated by the changes in the DTT parameters. The decreases in FA values indicate a decrease in the directionality of the PTT while the increases in MD and VN values indicate increases in the magnitude of water diffusion and the number of neural fibers of the PTT.^[26,27]^ Based on these results, we suggest that C/P in these patients mitigated some of the adverse conditions that were affecting the PTT within the PFC on both the OP and contra-OP sides, thereby inducing a relatively rapid decrease in the directionality and increase in water diffusion and fiber number of the PTT.^[4,5,9,18]^

Several studies have reported PFC changes can occur concurrently with recovery of cognitive impairment after C/P.^[15–20]^ In 2005, Maeshimaa et al, using single-photon emission computed tomography, demonstrated that the recovery of cerebral blood flow in both frontal regions in a patient with traumatic brain injury (TBI) who showed a reduction of cognitive impairment after C/P.^[15]^ In 2014, Coelho et al reported on a patient with TBI who presented with increased cerebral blood flow on CT perfusion and reduction of cognitive impairment following C/P.^[16]^ Subsequently, Sebastianelli et al (2015) reported on a stroke patient who revealed restoration of hypoperfusion of the DLPFC on electroencephalography concurrent with a reduction of cognitive impairment following C/P.^[17]^ In 2018, Jiang et al demonstrated improvements to cerebral blood flow to and cerebral blood volume of the frontal lobe after C/P in patients with TBI.^[18]^ In 2019, Panwar et al demonstrated improvement of cerebral blood flow of the OFC on CT perfusion in patients with TBI who showed improved cognitive function after C/P.^[19]^ In 2020, by applying MRI-based pseudo-continuous arterial spin-labeling perfusion imaging, Woo et al reported on a TBI patient with increased cerebral blood flow to the frontal lobe concurrent with the reduction of cognitive impairment after C/P.^[20]^Since the development of DTT, a few studies have reported on changes to neural tracts following C/P.^[27,28]^ Jang et al (2017) reported the recovery of an injured corticobulbar tract on the contra-OP side in a stroke patient who also showed recovery of dysphagia after C/P.^[27]^ Recently, restoration of the corticoreticulospinal tract in both hemispheres was concurrent with motor recovery of the affected leg after C/P in a stroke patient.^[28]^ However, to the best of our knowledge, this is the first study to use DTT to demonstrate changes in the PTT that were concurrent with the reduction of cognitive impairment following C/P in stroke patients. However, some limitations of this study should be considered. First, DTT analysis may underestimate or overestimate fiber tract status due to regions of fiber complexity and crossing fiber.^[30]^ Second, because this is a case-based report, generalizations based on this study should be limited; therefore, further studies involving large numbers of patients and comparing with a group without C/P or a group which does not show improvement of cognitive dysfunction following C/P are necessary.

## 5 Conclusion

In conclusion, by using DTT and MMSE, we demonstrated structural changes in the PTT along with concomitant reductions of cognitive impairments following C/P in 3 women with hemorrhagic stroke. Our results suggest that C/P can affect the state of the PTT on both the OP and contra-OP sides.

## Author contributions

EBC: study design, writing, and critical revision of manuscript for intellectual content.

CHC: study design, and development, and critical revision.

SHJ: study concept and design, manuscript development, writing, funding, and critical revision of manuscript for intellectual content.

**Conceptualization:** Sung Ho Jang.

**Data curation:** Chul Hoon Chang.

**Formal analysis:** Eun Bi Choi.

**Methodology:** Eun Bi Choi, Chul Hoon Chang.

**Resources:** Sung Ho Jang.

**Supervision:** Sung Ho Jang.

**Writing – original draft:** Eun Bi Choi, Sung Ho Jang.

**Writing – review & editing:** Sung Ho Jang.
